# *Wolbachia* Feminises a Spider Host With Assistance From Co‐Infecting Symbionts

**DOI:** 10.1111/1462-2920.70149

**Published:** 2025-07-10

**Authors:** Virginija Mackevicius‐Dubickaja, Yuval Gottlieb, Jennifer A. White, Matthew R. Doremus

**Affiliations:** ^1^ Koret School of Veterinary Medicine, The Robert H. Smith Faculty of Agriculture, Food and Environment The Hebrew University Rehovot Israel; ^2^ S‐225 Agriculture Science Center N, Department of Entomology University of Kentucky Lexington Kentucky USA; ^3^ Department of Entomology University of Illinois at Urbana‐Champaign Urbana Illinois USA

**Keywords:** co‐infection, feminisation, heritable symbiosis, reproductive manipulation, spiders, *Wolbachia*

## Abstract

Arthropods commonly harbour maternally‐transmitted bacterial symbionts that manipulate host biology. Multiple heritable symbionts can co‐infect the same individual, allowing these host‐restricted bacteria to engage in cooperation or conflict, which can ultimately affect host phenotype. The spider *Mermessus fradeorum* is infected with up to five heritable symbionts: *Rickettsiella* (R), *Tisiphia* (T), and three strains of *Wolbachia* (W1‐3). Quintuply infected spiders are feminised, causing genetic males to develop as phenotypic females and produce almost exclusively female offspring. By comparing feminisation across nine infection combinations, we identified a *Wolbachia* strain, W1, that is required for feminisation. We also observed that spiders infected with both W1 and W3 produced ~10% more females than those lacking W3. This increase in feminisation rate does not seem to be due to direct changes in W1 titre, nor does W1 titre correlate with feminisation rate. Instead, we observed subtle titre interactions among symbionts, with lower relative abundance of R and T symbionts in strongly feminised infections. This synergistic effect of co‐infection on *Wolbachia* feminisation may promote the spread of all five symbionts in spider populations. These results confirm the first instance of *Wolbachia*‐induced feminisation in spiders and demonstrate that co‐infecting symbionts can improve the efficacy of symbiont‐induced feminisation.

## Graphical abstract

Terrestrial arthropods often host maternally‐transmitted bacterial symbionts that modify host biology to increase production of infected females, with some symbionts feminising genetic males to develop as phenotypic females. We demonstrate that a strain of the symbiont *Wolbachia* is required for feminisation in the spider, *Mermessus fradeorum* . Co‐infection with four other symbionts increases feminisation penetrance, possibly via subtle shifts in the relative abundance of each symbiont within the host. Our findings suggest that co‐infecting symbionts can have synergistic effects on symbiont‐induced feminisation.

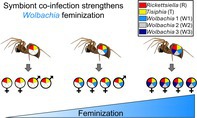

## 1 Introduction

Maternally‐transmitted bacterial symbionts commonly infect terrestrial arthropods. These host‐restricted heritable symbionts play major roles in the biology of their hosts, often manipulating host reproduction to favour infected females that transmit the symbiont (Doremus and Hunter 2020). Heritable symbionts, such as the common symbiont *Wolbachia*, can use several different strategies to manipulate host reproduction ranging from inducing asexual reproduction to causing mating incompatibilities between differentially infected hosts or biasing host development to favour females (Doremus and Hunter 2020). Some heritable bacteria can also bias host development to favour females by feminising genetic males, causing them to develop instead as phenotypic females capable of reproducing and transmitting the symbiont (Doremus and Hunter 2020; Werren et al. 2008).

*Wolbachia* are responsible for feminising a diverse range of hosts including isopods (Bouchon et al. 1998), lepidopterans (Hiroki et al. 2002; Sugimoto and Ishikawa 2012), and planthoppers (Negri et al. 2006). Feminisation is also implicated in *Wolbachia*‐induced parthenogenesis of some hymenopteran parasitoids (Fricke and Lindsey 2024). Feminising symbionts can alter the dynamics of host populations by causing female‐biased sex ratios (Bourtzis and Miller 2008; Hatcher et al. 1999; Kageyama et al. 2012), male rarity, and in extreme cases, may even influence the evolution of host sex determination systems (Bourtzis and Miller 2008; Cordaux et al. 2011; Cordaux and Gilbert 2017; Sugimoto and Ishikawa 2012). Despite affecting a range of arthropod hosts, *Wolbachia* feminisation remains comparatively less characterised compared to its other forms of reproductive manipulation (Beckmann et al. 2017; LePage et al. 2017; Shropshire et al. 2018; Perlmutter et al. 2019; Fricke and Lindsey 2024).

The presence of multiple heritable symbiont strains within the same host provides an opportunity for inter‐strain interactions that can favour certain symbiont combinations over others (Doremus and Oliver 2017; Leclair et al. 2017; Peng et al. 2023; Weldon et al. 2020; Zhu et al. 2012). These interactions include possible conflict over resources or access to intracellular niches within the host (Goto et al. 2006; Yang et al. 2020), or cooperation to facilitate their transmission (Peng et al. 2023). Symbiont co‐infections can alter symbiont titre (Goto et al. 2006; Kondo et al. 2005; McLean et al. 2018; Yang et al. 2020), tissue tropism (Goto et al. 2006), transmission rate (Rock et al. 2018), and metabolic complementation (Peng et al. 2023), all of which can ultimately influence the penetrance of symbiont‐induced phenotypes and the stability of symbiont co‐infections.

Symbiont co‐infections are particularly common in spiders, which often host heritable symbiont communities whose complexity rivals that of their better‐studied insect cousins (Goodacre et al. 2006; Vanthournout and Hendrickx 2015; Zhang et al. 2018; White et al. 2020). For example, North American populations of *Mermessus fradeorum* , a cosmopolitan dwarf spider, are variably infected with up to five heritable symbionts: *Rickettsiella*, *Tisiphia* (formerly *Rickettsia*; Davison et al. 2022), and three strains of *Wolbachia* (Curry et al. 2015; Rosenwald et al. 2020). The most common symbiont, *Rickettsiella*, usually infects > 99% of spiders in Central US populations and causes a form of reproductive sabotage called cytoplasmic incompatibility (Rosenwald et al. 2020). The next most common symbiont combination (symbiotype) is all five symbionts co‐infecting the same spider; these quintuply infected spiders produce strongly female‐biased offspring broods (Curry et al. 2015; Rosenwald 2020; Rosenwald et al. 2020). This female bias is caused by feminisation of genetic males by one or more of the co‐infecting symbionts; approximately half of eggs in these lines have the chromosomal content of genetic males (2*n* = 24, females are 2*n* = 26). Furthermore, these spiders do not exhibit extreme egg mortality indicative of other female biasing phenotypes like male‐killing (Curry et al. 2015). The *M. fradeorum* system provides an excellent opportunity to study multiple forms of reproductive sabotage in the same host and to learn more about the biology of heritable symbioses of spiders (Goodacre et al. 2006; Vanthournout and Hendrickx 2015; Zhang et al. 2018; White et al. 2020) but further research requires identifying which of the symbiont(s) are responsible for feminisation.

By comparing offspring sex ratios among spiders harbouring nine different symbiotypes, we identified a strain of *Wolbachia* (W1) as necessary for feminisation in *M. fradeorum* . Co‐infection with certain symbionts synergistically improved W1 feminisation, which may reinforce co‐transmission of the five‐member symbiont community in nature. We additionally sought to quantify symbiont titre in different symbiotypes using digital PCR because symbiont titre can both influence host phenotype and change in response to co‐infection. This method enables precise and absolute quantification of a given template by partitioning the sample into thousands of replications in a single reaction. We found that W1 numerically dominates the heritable symbiont community and is consistently the most abundant symbiont when present. Despite the overall higher titre of W1 compared to other symbionts in this system, W1 titre was not correlated with feminisation rate. Instead, the relative abundance of R and T symbionts was reduced in strongly feminised symbiotypes, suggesting that improved *Wolbachia* feminisation may arise from subtle interactions among co‐infecting symbionts. These results confirm the first instance of *Wolbachia*‐induced feminisation in a spider, inform our understanding of *M. fradeorum* feminisation, and demonstrate that symbiont co‐infections can have synergistic effects that increase the penetrance of symbiont‐conferred feminisation.

## 2 Experimental Procedures

The *M. fradeorum* cultures used in this study were initially collected from alfalfa ( *Medicago sativa* ) fields in Kentucky, USA, and subsequently maintained in the laboratory. Spiders were kept at 20°C in individual 4 cm diameter rearing cups with moistened plaster at the bottom for humidity control (Rosenwald et al. 2020). Immature spiders were fed collembola ( *Sinella curviseta* ) until they neared adulthood; thereafter, they were fed one wingless *Drosophila melanogaster* twice a week.

North American field populations of *M. fradeorum* are naturally infected with up to five heritable symbionts: *Rickettsiella* (R), *Tisiphia* (T), and three strains of *Wolbachia* (W1, W2, W3; Rosenwald et al. 2020). For simplicity, we will refer to symbionts by their letter abbreviations. To identify which of these five symbionts contribute to feminisation, we used a panel of nine symbiotypes including quintuply infected (RTW123), quadruply infected (RW123, RTW12, RTW23), triply infected (RTW1, RTW2), doubly infected (RT), singly infected (R), and uninfected spiders. All infected symbiotypes included *Rickettsiella* because we were unable to generate or maintain lineages that lost *Rickettsiella* without losing the other symbionts. These combinations included both naturally occurring symbiotypes and experimental symbiotypes generated via past antibiotic administration (Curry et al. 2015; Rosenwald et al. 2020). Antibiotic treatments consisted of misting developing spiders with a fine spray of tetracycline (0.1%) and ampicillin (0.1%) until sub‐adulthood (Rosenwald et al. 2020). All lines produced via antibiotic treatments were maintained in the lab for at least five generations prior to use in experiments. Symbiont infection status was confirmed using diagnostic PCR (Data S1).

We mated 8–12‐week‐old female spiders representing the nine symbiotypes with uninfected male spiders. After females laid one egg mass, they were held for 5 days in a 1.5 mL microcentrifuge tube to void their gut contents and then were stored in 95% ethanol at −20°C until we extracted their DNA using DNeasy Blood and Tissue kits (Qiagen) following the manufacturer's protocol. To estimate sex ratio, we randomly separated up to eight siblings per egg mass into individual 4 cm cups to avoid cannibalism. Egg masses that produced fewer than four offspring were removed from the experiment. These offspring were reared at 20°C and were first provided *S. curviseta* and then *D. melanogaster* as they developed. We determined offspring sex morphologically by the presence of enlarged pedipalps in males once spiders reached the penultimate immature stage. Using logistic regression with a quasibinomial distribution (R v 4.4.0, R Core Team 2024), we tested the effect of symbiont infection on spider sex ratio using a series of planned contrasts between infected spiders and uninfected control spiders. These contrasts included a full model comparing the entire set of eight infected symbiotypes and the uninfected control, as well as contrasts between each individual symbiotype and the control. We next used logistic regressions to compare feminisation rates between each feminised symbiotype and the quintuply infected line to see if the presence or absence of co‐infecting symbionts modified the level of feminisation.

We also assessed whether symbiont titre was related to feminisation. We conducted digital PCR (dPCR) assays to further confirm infection of experimental spiders and to quantify symbiont genome copies in feminised *M. fradeorum* samples using short specific primers and probes designed for symbiont and spider genes as specified in Table 1. We used Geneious Prime version 2021.2 software (https://www.geneious.com/updates) to design primers and probes, DINAMelt Web Server (http://www.unafold.org/Dinamelt/applications/two‐state‐melting‐folding.php) to predict hybridisation and folding of the amplicons, and BLAST Web Server (https://blast.ncbi.nlm.nih.gov/Blast.cgi) to test primer specificity. Primers and probes were obtained from Biolegio BV (Nijmegen, Netherlands) and were dissolved in TE buffer (Tris‐EDTA; 10 mM Tris base, 0.1 mM EDTA, pH 8.0) to make 100 μM stocks and further diluted with TE buffer to create 20 μM working solutions (stocks and working solutions stored at −20°C until use). TaqMan probes were synthesised by labeling the 5′ terminal nucleotide with a fluorophore and the 3′ terminal nucleotide with a dark quencher, Black Hole Quencher 1 (BHQ1) or Black Hole Quencher 2 (BHQ2). Probes and primers were tested for specificity using diagnostic PCR and quantitative PCR (diagnostic and qPCR details included in Data S1).

**TABLE 1:** Primers and probes used for digital PCR.

Target	Target gene	Primer/probe name	Sequence 5′ to 3′	Amplicon length (bp)
*M. fradeorum*	*18S rRNA*	18S_1375F	5′‐CATGGAGCTTGCGGTTCAAT‐3′	170
		18S_1544R	5′‐AGAGCCTCGTCCGTTATCAG‐3′	
		18S_P	5′‐FAM‐TCCAGGCCAGGACACAGGGAGGATT‐BHQ1‐3′	
*Rickettsiella*	*recA*	recA_612F	5′‐GGAAACAACAACGGGTGGTA‐3	120
		recA_811R	5′‐CACCGAGTCGACAAATACCC‐3′	
		recA_P	5′‐ROX‐ACGCGCTCAAGTTTTATGCTTCCGTACGT‐BHQ2‐3′	
*Tisiphia*	*rpoB*	rpoB_813F	5′‐AGCCTTGATGGGGTCAAACA‐3′	121
		rpoB_933R	5′‐GGCTACAACTGAAACCCCAGA‐3′	
		rpoB_P	5′‐Atto550‐TGCAACGTCAGGCTGTCCCGCTT‐BHQ2‐3′	
*Wolbachia*1	*wsp1*	wsp1_P	5′‐HEX‐AATGATACCAATGCTGCAGATGGTG‐BHQ1‐3′^a^	138
		wsp1/wsp2_F	5′‐TGATGTTGAAGGGCTTTACTCACA‐3′	
*Wolbachia*2	*wsp*2	wsp1/wsp2_R	5′‐GGCATATCTTCAATCGCTACATCG‐3′	
		wsp2_P	5′‐Cy5‐GCTGCTGAGACAAATGTTGCAGATA‐BHQ2‐3′^a^	
*Wolbachia*3	*wsp*3	wsp3_209F	5′‐GCCTATCACTCCATACGTTGGT‐3′	118
		wsp3_326R	5′‐ACCAGCTTTTACTTGACCAGCAA‐3′	
		wsp3_P	5′‐FAM‐AACCGCTGTGAATGATCAAAACAGT‐BHQ1‐3′^a^	

^a^ With XS modification to increase annealing temperature (https://www.biolegio.com/products/xs‐probes).

We performed the detection and amplification of the spider *18S rRNA* gene separately from symbiont genes using Qiagen QIAcuity Probe PCR Kit and QIAcuity One‐plate digital PCR instrument (QIAcuity One, 5plex instrument, Qiagen, Germany). The dPCR mixture for a single reaction was 10 μL of 4× Probe QIAcuity PCR Master Mix, 0.8 μM final concentration of each primer, 0.4 μM final concentration of probe, 12/1.2 ng of samples gDNA for symbiont/18S rRNA gene, respectively, and RNase‐free water for a total reaction volume of 40 μL. Once prepared, the reaction mixtures were transferred to QIAcuity Nanoplate 26 k 24‐well and sealed with the QIAcuity Nanoplate Seal. We used the following dPCR cycling programme: 2 min at 95°C for initial heat activation, followed by 40 cycles of 95°C for 15 s for denaturation, and 60°C for 30 s for annealing and extension. All dPCR runs contained negative controls containing the same DNA background without the targeted sequence and/or DNA Elution buffer AE (10 mM Tris‐HCL, 0.5 mM EDTA, pH 9, Qiagen), and the same two quintuply infected positive controls to enable data comparison across multiple nanoplates. After the reaction finished, the signal was quantified, and we set thresholds manually to separate two populations: positive partitions with increased fluorescence intensity and negative partitions with baseline signal only. Reactions containing below 100 random unexpected positive partitions of the symbiont gene with a high confidence interval (CI) were considered negative for that symbiont. We calculated gene copy number per μL for each gene using the integrated QIAcuity Software Suite. To adjust for the gDNA input amount (12 ng of sample for symbiont detection, 1.2 ng of sample for spider gene detection for each dPCR reaction), we multiplied *18S rRNA* copies/μL by this gDNA dilution factor. Normalised symbiont titers (normalised symbiont titre = symbiont gene copy number/*18S rRNA* gene copy number) for every symbiont were compared across feminised symbiotypes using Kruskal–Wallis tests, followed by pairwise Mann–Whitney *U*‐tests to determine differences between specific pairs of symbiotypes in R (R v 4.4.0, R Core Team 2024). We also tested for a correlation between W1 titre and feminisation rate using Spearman's correlation test in R (R v 4.4.0, R Core Team 2024).

The initial crossing experiment showed an effect of symbiont co‐infection on feminisation rate, with spiders infected with all five symbionts showing increased feminisation rates compared to spiders infected with subsets of fewer symbionts. To confirm that the observed differences in feminisation between RTW123 and RTW1 spiders were replicable, we performed an additional experiment comparing the sex ratio among uninfected (*n* = 12), triply infected (RTW1; *n* = 12), and quintuply infected (RTW123; *n* = 12) spiders. As in the previous experiment, eight‐week‐old female spiders were mated with uninfected males, allowed to lay one egg mass, starved for 5 days then stored in 95% ethanol at −20°C prior to DNA extraction. For this set, symbiont infection status was confirmed with diagnostic PCR (Data S1). We estimated feminisation rates by morphologically characterising the sex of 9 randomly selected offspring as in the first assay. We then compared feminisation rates across these three infection types using logistic regression with a quasibinomial distribution in R (R v 4.4.0, R Core Team 2024).

## 3 Results

### 3.1 Co‐Infection Modifies the Efficacy of *Wolbachia* 1‐Induced Feminisation

**FIGURE 1:**
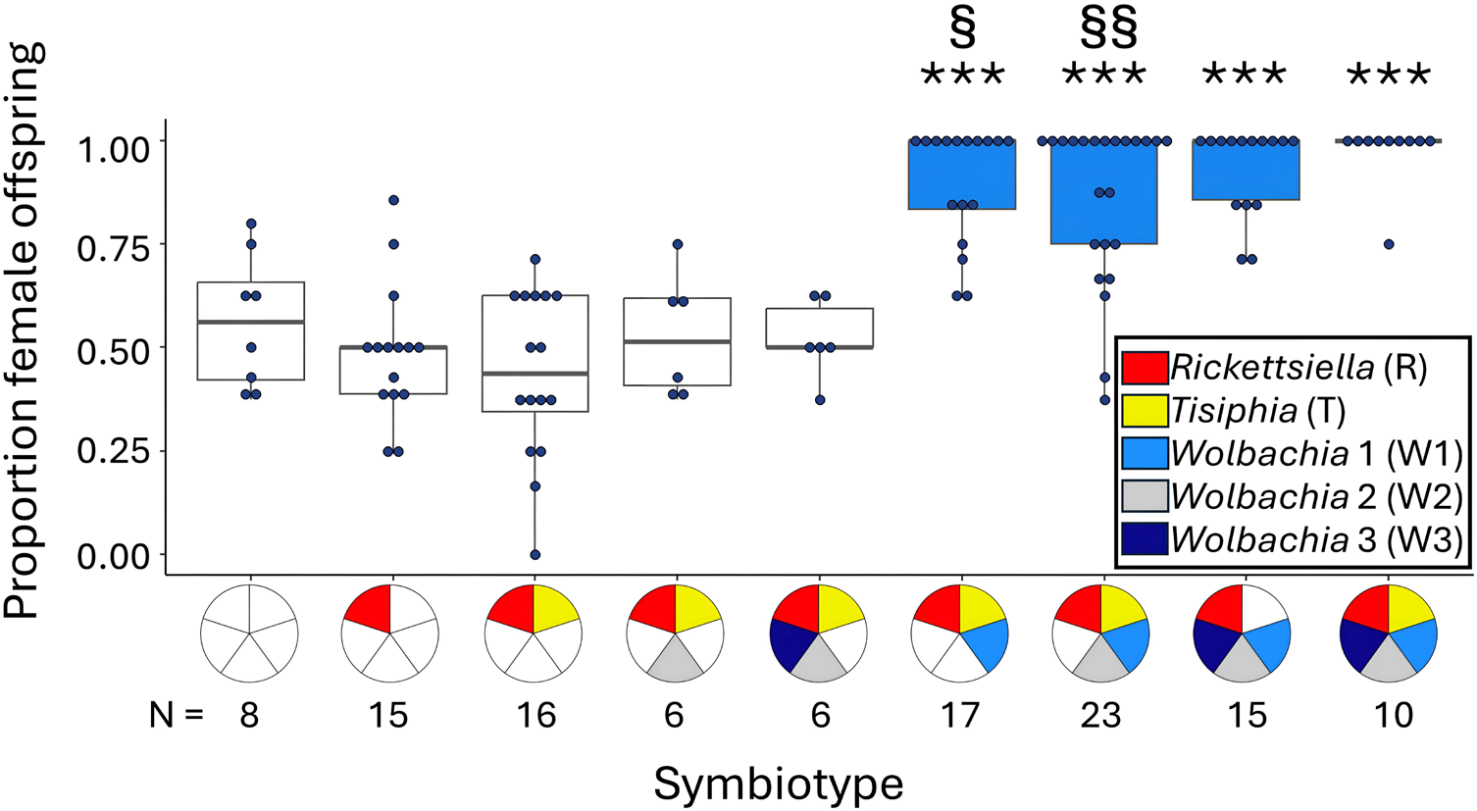
Proportion of female offspring produced by female *M. fradeorum* infected with different symbiont combinations. Symbiotypes are represented with coloured pie charts on the x‐axis, with each portion of the chart representing a symbiont as indicated in the key. Significant differences in proportion of female offspring compared to uninfected females are denoted with asterisks (***p < 0.001). Significant differences in proportion female offspring of different W1 infection combinations compared to RTW123 spiders are denoted by the symbol § (§*p* < 0.05, §§*p* < 0.01). Box colours represent W1 infection, with infections lacking W1 coloured white and infections including W1 coloured blue. Sample sizes are listed below infection status.

Offspring sex ratio (Figure 1) varied significantly across spiders harbouring different symbiotypes (Δdeviance = 189.22, df = 8, *p* < 0.001). Compared to uninfected spiders, which generally produce an even sex ratio, quintuply infected female spiders (RTW123) produced almost exclusively female offspring as expected (Δdeviance = 36.34, df = 1, *p* < 0.001; Curry et al. 2015). Other symbiotypes that contained W1 (RTW1, RTW12, RW123) also exhibited strongly female‐biased sex ratios compared to uninfected spiders (Δdeviance_RTW1_ = 24.89, df = 1, *p* < 0.001; Δdeviance_RTW12_ = 20.61, df = 1, *p* = 0.001; Δdeviance_RW123_ = 30.82, df = 1, *p* < 0.001). Spiders lacking W1 (R, RT, RTW2) did not differ from uninfected controls (Δdeviance_R_ = 0.64, df = 1, *p* = 0.345; Δdeviance_RT_ = 0.65, df = 1, *p* = 0.404; Δdeviance_RTW2_ = 0.06, df = 1, *p* = 0.768). Strikingly, spiders infected with all symbionts except W1 also were not feminised compared to uninfected spiders (Δdeviance_RTW23_ = 0.14, df = 1, *p* = 0.607), confirming that W1 is essential for feminisation and is likely the sole inducer of this phenotype in *M. fradeorum* .

However, not all combinations containing W1 were equal. Quintuply infected spiders produced a higher proportion of female offspring than either RTW1 (Δdeviance = 4.46, df = 1, *p* = 0.03) or RTW12 spiders (Δdeviance = 8.43, df = 1, *p* = 0.004). Feminisation rates of RW123 spiders were similar to those of quintuply infected spiders (Δdeviance = 1.56, df = 1, *p* = 0.212), indicating that certain combinations of co‐infecting symbionts can influence the penetrance of W1 feminisation. When W1‐infected spiders also harboured W3 (RW123, RTW123), their feminisation rates were ~10% higher than those that lacked this additional *Wolbachia* strain.

A second, more targeted assay compared feminisation rates among uninfected, RTW1, and RTW123 spiders also found stronger feminisation in RTW123 than in RTW1 spiders (Figure S1; Δdeviance = 8.01, df = 1, *p* = 0.029). As in the first experiment, feminisation was ~10% stronger in RTW123 spiders than in RTW1 spiders, reinforcing the finding that co‐infection by other symbionts strengthens feminisation.

### 3.2 Symbiont Titers Show Limited Change Across Infection Types and Feminisation Rates

**FIGURE 2:**
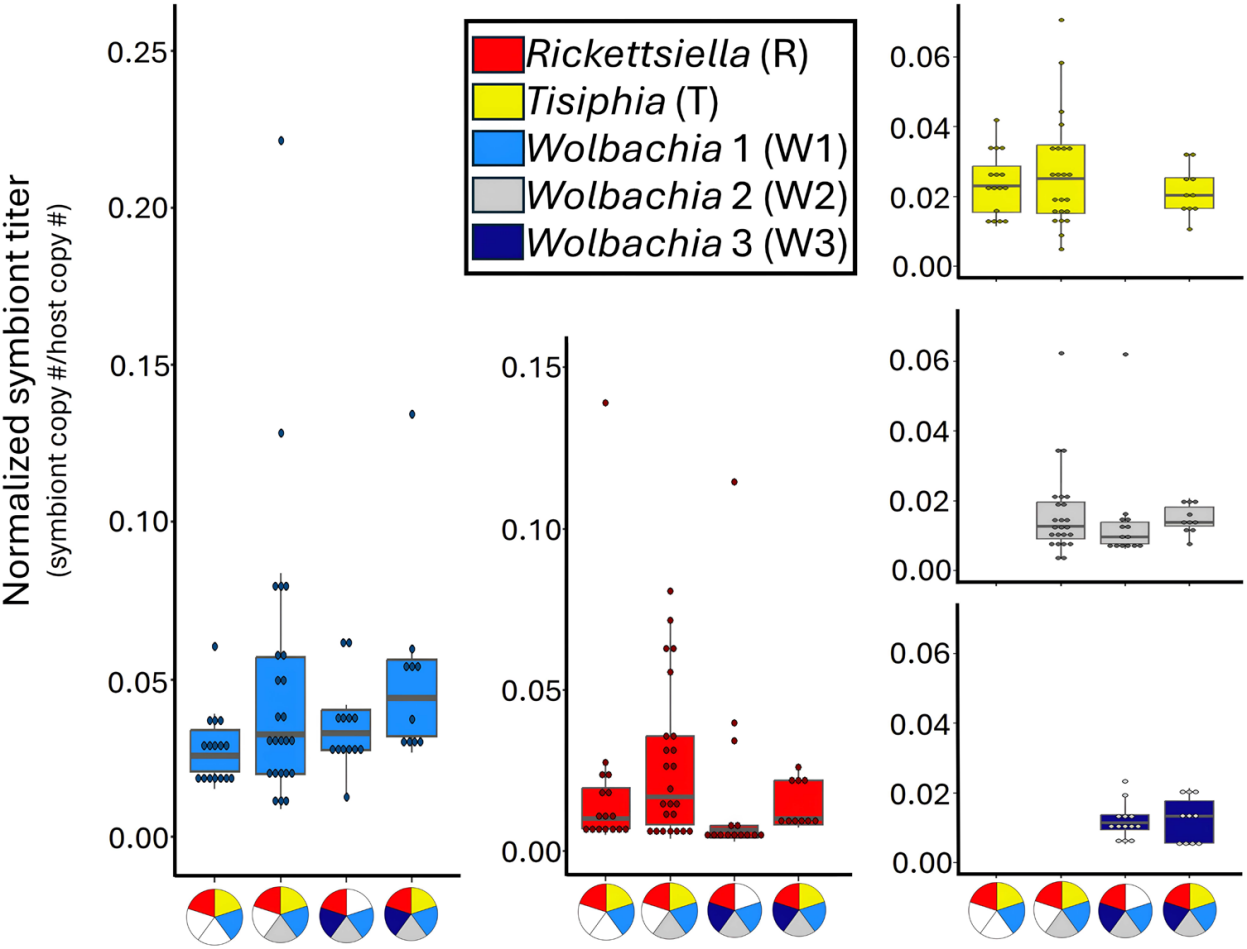
Symbiont titers estimated with dPCR normalised to host 18 s rRNA in spiders infected with feminising symbiotypes. Inset key indicates which symbiont is depicted in which panel and which symbiotypes are represented by pie charts on the *x*‐axes. Symbiont genes used in dPCR were *recA* (*Rickettsiella*), *rpoB* (*Tisiphia*), and *wsp* (*Wolbachia* 1‐3). Normalised symbiont titre was compared between infection types using multiple pairwise Mann–Whitney *U*‐tests with Benjamini–Hochberg corrected *p*‐values. Letters represent significantly different normalised titers (*p* < 0.05).

In the main experiment, normalised W1 titers only marginally changed across the four feminised symbiotypes (K–W *χ* ^2^ = 6.38, df = 3, *p*‐value = 0.09). Pairwise comparisons showed W1 titre to be lower in RTW1 spiders than RTW123 spiders (Mann–Whitney *U*‐test *p*‐value = 0.036; Figure 2), with other symbiotype combinations having intermediate W1 titers (MWU *p*‐value = 0.278–0.870 for all comparisons). *Rickettsiella* titre showed a significant change across symbiotypes (Kruskal–Wallis *χ* ^2^ = 7.98, df = 3, *p*‐value = 0.05), possibly driven by reduced R titre in RW123 spiders (Figure 2). Yet pairwise comparisons among R‐containing symbiotypes did not show significant differences (MWU *p*‐value = 0.092–0.623 for all comparisons). The titers for T (K–W *χ* ^2^ = 0.79, df = 2, *p*‐value = 0.67), W2 (K–W *χ* ^2^ = 3.07, df = 2, *p*‐value = 0.21), and W3 (K–W *χ* ^2^ = 0.01, df = 1, *p*‐value = 0.91) did not change across their respective infected symbiotypes (Figure 2). A Spearman correlation test did not support a correlation between sex ratio and normalised W1 titre (Figure S2; Spearman's *r*_*s*_ = 0.026, *p*‐value = 0.84).

### 3.3 *Wolbachia* 1 Relative Abundance is Highest and Stable Across Feminised Infection Types

**FIGURE 3:**
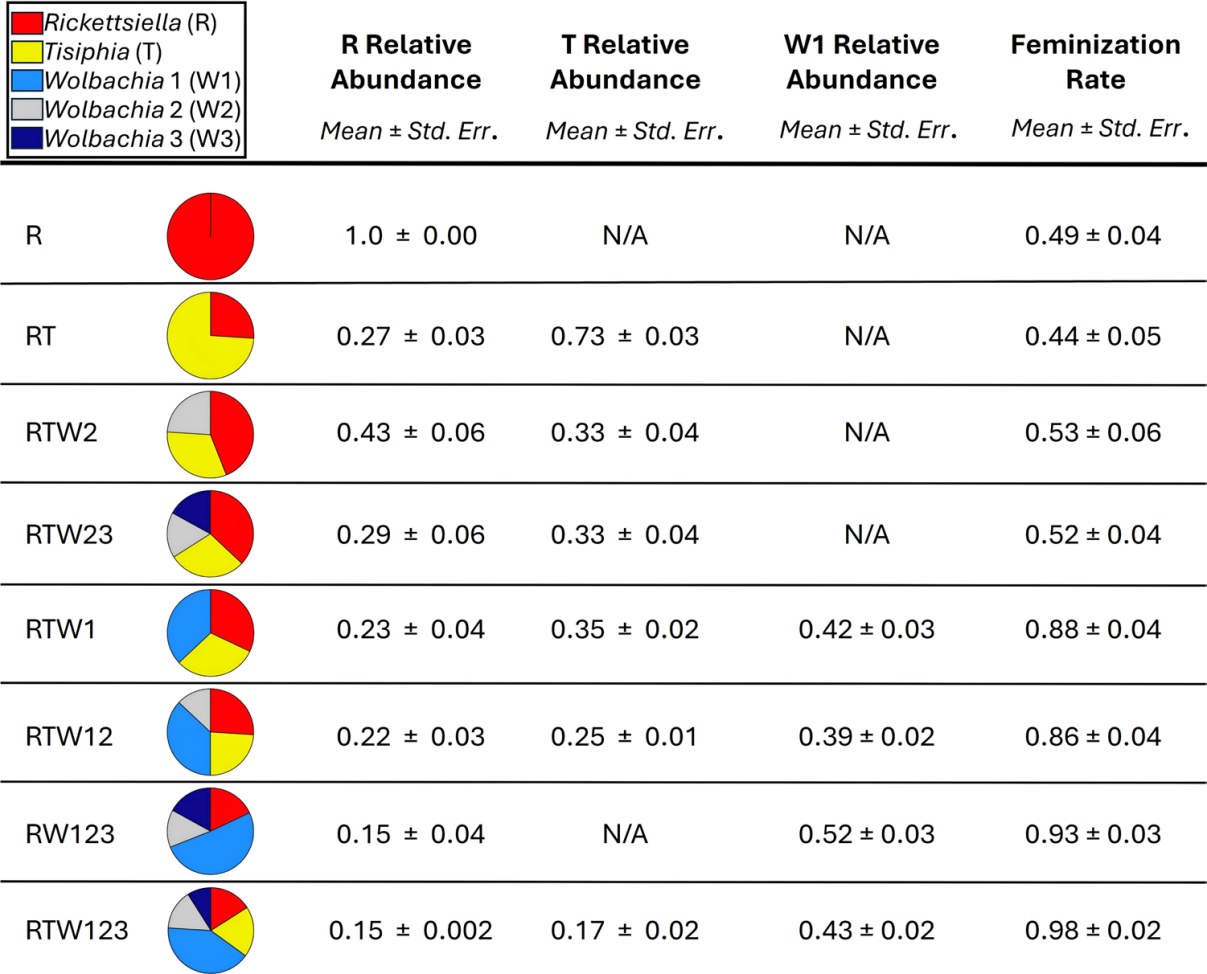
Mean relative abundance for R, T, and W1 in spiders with different infection combinations and feminisation rates. The relative abundance of symbionts is expected to fall as the number of symbiont strains increases, but *Wolbachia* 1 maintains the highest proportion regardless of other members of the symbiont community. R and T represent the highest proportion when *Wolbachia* 1 is not present, with their lowest relative abundances occurring in symbiotypes with the highest feminisation rates.

Despite relatively subtle changes in symbiont titre, we did observe qualitative changes in the relative abundance of some symbiont symbiotypes (Figure 3). The relative abundance of each symbiont is expected to fall as more symbionts are added to the community. For example, W3 is relatively more abundant in quadruple infections (RW123, RTW23) compared to the quintuple infection (Figure 3). Yet the abundance of W1 relative to other symbionts remained stable across infections, with W1 comprising ~40% to 50% of the total symbiont titre. Other symbionts, most notably R and T, exhibited a marked reduction in relative abundance in the most feminised symbiotypes, RW123 and RTW123. *Rickettsiella* comprises ~30% to 40% of the symbiont titre in co‐infected symbiotypes lacking W1, but its proportional representation drops to < 20% of the total titre in RW123 and RTW123 spiders. Those symbiotypes also featured a similar reduction in *Tisiphia* abundance, dropping from ~30% to < 20% of total symbiont titre. These changes suggest that the structure of this heritable symbiont community undergoes a substantial shift favouring *Wolbachia* 1 when this symbiont is present.

## 4 Discussion

We found that one *Wolbachia* strain (W1) is required for feminisation in *M. fradeorum* (Figure 1 **)**. Spiders harbouring W1 exhibited female‐biased sex ratios, while those lacking W1 did not. As we did not evaluate the phenotype of spiders singly infected with W1, it remains possible that *Rickettsiella* (and/or other symbionts like *Tisphia*) might also play a direct role in feminisation when W1 is present. Despite numerous attempts, we have been unable to generate symbiotypes that do not include *Rickettsiella* in this system. Our results therefore highlight that W1 is a necessary component for feminisation of *M. fradeorum* , but do not yet show that it is sufficient for feminisation on its own.

We also found a synergistic effect of co‐infection on *Wolbachia* feminization, with spiders harbouring two symbiotypes, RTW123 and RW123, exhibiting ~10% stronger feminization than other feminised symbiont combinations (Figures 1 and S1). This synergistic increase in feminization may involve direct changes in the feminising *Wolbachia* titre, as both W1 titre and feminization rate were increased in RTW123 spiders relative to the more variably feminised RTW1 spiders (Figure 2). However, because feminization rates were generally high in this system across all symbiotypes bearing W1, we ultimately did not find a correlation between W1 titre in adult females and their feminization rate (Figure S2). Symbiont titre at other stages of development (e.g., embryogenesis) might also be more reflective of realised feminization rates (Doremus et al. 2019, 2022; Herran et al. 2020). Additionally, while *Wolbachia* titre is generally thought to correspond with feminising phenotypic penetrance (Rigaud et al. 2001; Narita et al. 2007; Herran et al. 2020), there are instances of low‐titre *Wolbachia* capable of inducing strong manipulative phenotypes (Richardson et al. 2019). It is possible that W1 only requires a low titre threshold for reliable feminization induction, which would further limit our ability to identify a correlation between titre and phenotype expression.

The influence of titre on feminisation is seemingly subtle in this system and could also involve changes in the composition of the entire symbiont community (Figure 3), rather than the normalised titre of any one symbiont. The composition of the symbiont community shifts in feminised spiders to favour W1, which seems to dominate the symbiont community when present. This shift comes largely at the expense of the relative abundance of *Rickettsiella* and *Tisiphia*, with the remaining *Wolbachia* strains showing more limited changes in relative abundance across symbiotypes (Figure 3). The variation in symbiont community composition hints at potential competitive interactions among symbionts that seemingly favour *Wolbachia* 1.

This web of symbiont interactions may alter W1 biology in other ways beyond shifting its titre, such as influencing W1 localisation or gene expression. For example, many bacteria regulate their gene expression through quorum sensing in response to bacterial cell density, including that of other bacterial strains and species (Miller and Bassler 2001). Hypothetically, co‐infecting symbionts could also indirectly affect feminisation by altering host biology, perhaps by altering host developmental time during critical life stages or by producing co‐factors that improve the efficacy of W1‐encoded feminising factors.

The feminising capabilities of W1 should allow it to spread through spider populations regardless of its co‐infecting partners, yet W1 is usually observed in quintuple infections or alongside W3 in nature (Rosenwald et al. 2020). This infection pattern is suggestive of a synergistic link between these symbionts (Rock et al. 2018; Rosenwald et al. 2020; Peng et al. 2023). The augmentation of W1 feminisation by co‐infecting symbionts, particularly W3 (Figures 1 and S1), may promote the transmission of the quintuple infection. This cooperative effect clearly benefits symbionts like W3, which do not seem to directly influence host reproduction and thus benefit from hitchhiking off improved W1 feminisation. However, it is not fully clear how this interaction benefits W1, as W1 is still able to induce feminisation without W3, albeit with slightly reduced efficacy. The quintuple co‐infection might additionally benefit from other synergistic effects, such as increased transmission rates, reduced infection costs of W1, and/or context‐dependent benefits like protection from natural enemies (Rock et al. 2018). Future experimental manipulations of co‐infection via symbiont transinfection will improve our understanding of the consequences of co‐infection in this system.

Despite their prevalence, interactions among cohabiting symbionts remain understudied. The prominence of certain symbiotypes in host populations suggests that some symbiont combinations provide a selective advantage over others that can reinforce their shared transmission and spread (Peng et al. 2023). The effect of symbiotype on *Wolbachia*‐induced feminisation in *M. fradeorum* provides an example of synergy among symbionts. Identification of this effect, and the characterisation of the feminising symbiont of *M. fradeorum* , are essential steps in understanding the dynamics that shape the heritable symbiont communities of arthropods.

## Author Contributions

V. M‐D.: conceptualization, writing – original draft, writing – reviewing and editing, investigation, formal analysis, and methodology. Y.G.: conceptualization, writing – reviewing and editing, supervision, and funding acquisition. J.A.W.: conceptualization, writing – reviewing and editing, supervision, and funding acquisition. M.R.D.: conceptualization, writing – original draft, writing – reviewing and editing, visualization, investigation, data curation, methodology, project administration, and funding acquisition.

## Conflicts of Interest

The authors declare no conflicts of interest.

## Supporting information


**Data S1.** Supporting Information.

## Data Availability

The data that support the findings of this study are openly available in Dryad at DOI: 10.5061/dryad.f4qrfj77b.
